# Excision of Oral Submucous Fibrosis and Reconstruction with Full Thickness Skin Graft: A Case Study and Review of the Literature

**DOI:** 10.1155/2012/628301

**Published:** 2012-12-12

**Authors:** Ahmad Alshadwi, Ishwar Bhatia

**Affiliations:** ^1^Department of Oral and Maxillofacial Surgery, Boston Medical Center, Boston University, 100 East Newton Street, Boston, MA 02118, USA; ^2^Department of Oral and Maxillofacial Surgery, Henry Goldman School of Dental Medicine, Boston University, 100 East Newton Street, Boston, MA 02118, USA

## Abstract

Oral submucous fibrosis is a chronic debilitating disease characterized by gradually increasing fibrosis of the oral cavity and pharynx, mainly the buccal mucosa, resulting in trismus. The highest incidence of oral submucous fibrosis is seen in South India due to various deleterious habits. In spite of the numerous medical modalities employed in the management of oral submucous fibrosis, occasionally surgical intervention becomes inevitable. Various surgical approaches have been used to reconstruct the surgical defects following excision of fibrous bands. Full thickness skin grafts have been described in the literature with variable outcomes. In the present study a 38-year-old male presented with severe oral submucous fibrosis of the buccal mucosa, which was successfully treated and reconstructed using full thickness skin graft with stable functional result after one year of treatment. An integrated review of the literature regarding etiology, histopathology, diagnostic, and treatment modalities of the disease follows.

## 1. Introduction

Oral submucous fibrosis is commonly seen in the Indian subcontinent. The condition has a multifactorial origin but is commonly associated with chewing of areca nut (betal nut) habitually. This condition was first described by Schwartz in 1952 [1]. Oral submucous fibrosis is a chronic inflammatory reaction in the subepithelial tissue of the oral cavity. It results in increased fibroelastic changes by causing both increased collagen production and decreased collagen breakdown, accompanied by epithelial atrophy [2, 3]. The role of Arecoline and Tannis of areca nut causing juxtaepithelial hyalinization and muscle fibrosis is well described in the literature [4]. The clinical features such as excessive salivation, absent gustatory sensation, and limitation of mouth opening leads to difficulty in chewing, swallowing, articulation, and poor oral hygiene. The oral mucosa appears pale, dense, with vertical fibrotic bands extending to the anterior tonsillar pillars.

Various surgical techniques have been recommended to improve mouth opening in oral submucous fibrosis; we report a case of severe oral submucous fibrosis with limited mouth opening that has been released and reconstructed with full thickness skin graft. 

## 2. Case Report

**Figure 1:**
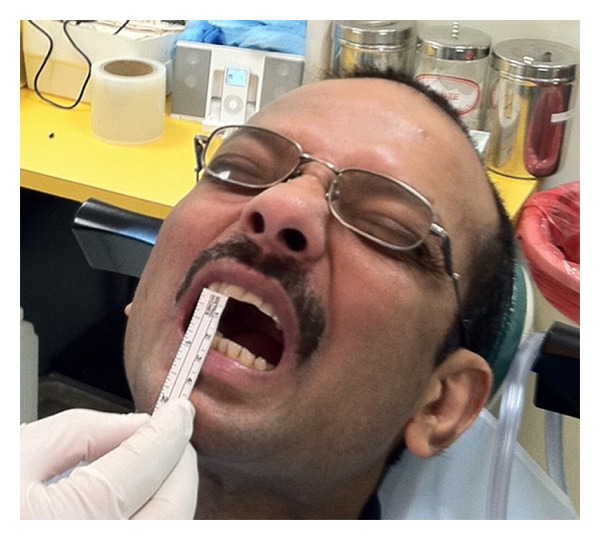
Preoperative view of the patient mouth opening.

**Figure 2:**
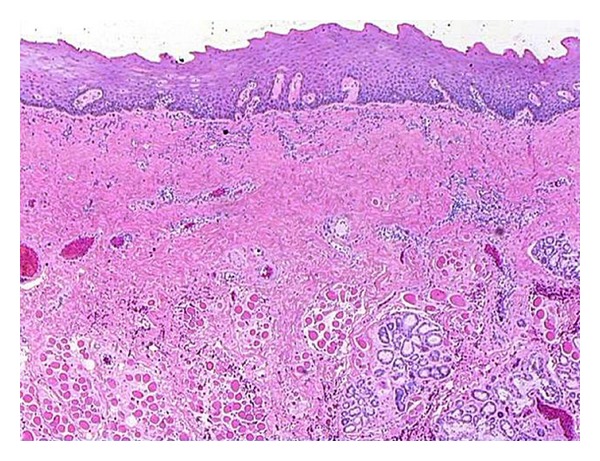
Low magnification H&E stained histopathological slide showing dense fibrosis of the stroma with infiltration to the skeletal muscular layer and minor salivary glands.

**Figure 3:**
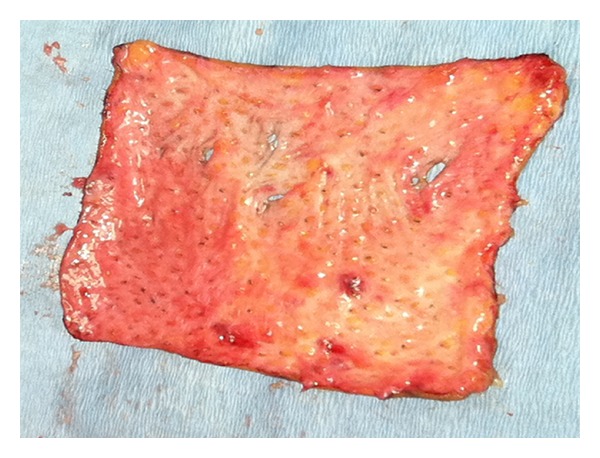
The harvested full skin graft measuring 7 × 7 cm.

**Figure 4:**
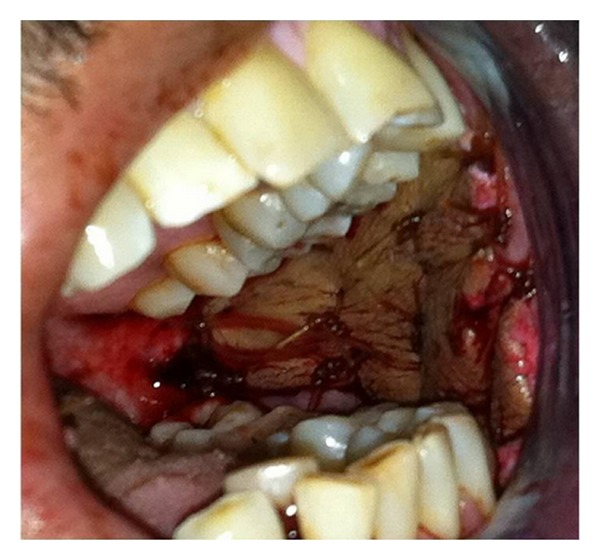
Intraoral views after suturing the graft to the buccal mucosa.

**Figure 5:**
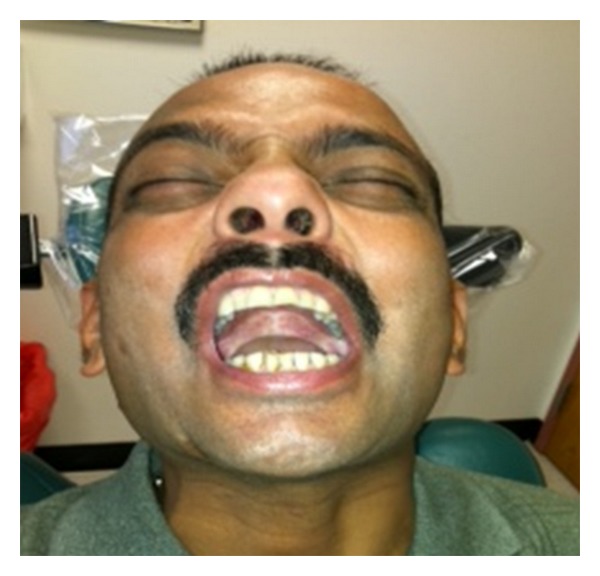
Sustained patient mouth opening at one year followup.

 A 38-year-old Indian male was referred to the Oral and Maxillofacial Surgery Clinic, Boston University Goldman School of Dental Medicine for the evaluation and management of progressive trismus. Patient presented with main complaint of inability to chew food and limited mouth opening. He reported that this has been going on with him for the last few years but became more concern for him just recently. His medical history was significant for hypertension, hypercholesterolemia, and benign prostatic hyperplasia which all are controlled by medications. Patient reported that he has been chewing pan of Indian tobacco for the last 15 years. Clinical exam showed no lymphadenopathy, swelling, or asymmetry. Limited mouth opening at 15 mm and cranial nerves exam is unremarkable except for alter sensation in the buccal branch of V 3 bilaterally (Figure 1). Intraoral exam showed poor oral hygiene, multiple carious teeth. Palpable severe fibrous bands in buccal mucosa from just inside the commeasure of the mouth up to the pterygomandibular raphe were felt bilaterally. Soft palate, tongue and floor of mouth were not involved. The fibrosis has resulted in pale-appearing mucosa. An incisional biopsy was taken for histopathological evaluation which confirmed the diagnosis of submucous fibrosis infiltrating the muscular layer (Figure 2), after discussion of the clinical findings and treatment options with the patient. The patient was taken to the operating room where he was placed on the surgical table hooked to the anesthesia, cardiac monitors, and pulse oximeter. Next patient was induced via intravenous general anesthesia and intubated with no complications. Local anesthesia was infiltrated in the intended surgical areas. Using a transoral approach a blade number 15 was utilized for the surgical excision of the fibrous bands and protecting underlying vital structures. Surgical dissection was carried out to the level of the buccinator muscle. After controlling bleeding the wound edges were prepared for the graft placement. Next attention was made toward the right thigh from which a 7 × 7 cm full thickness skin graft was harvested (Figure 3); using sharp dissection, was done to the level of the subcutaneous fat. Electrocautery was use to ensure homeostasis and appropriate pressure was placed on the donor site. After that the graft was cut to custom fit each defect and multiple perforations were done throughout the graft to ensure rapid revascularization and prevent hematomas from developing. Next the grafts were sutured into place using 3′0 Chromic Gut restorable sutures in simple interrupted fashion (Figure 4). Mouth opening was measured toward the end of the procedure and was recorded at 32 mm. After that the surgical area was irrigated and patient was extubated and transferred to the recovery room in stable condition. Postoperatively the patient was placed on full liquid diet for two weeks and one week supply of Motrin 400 mg, Percocet 5/325 mg, Keflex 250 mg, Medrol dose pack. Patient was followed regularly and on the second week his mouth opening was noted to decrease to 25 mm at which a course of total of 80 mg of Triamcinolone intramuscular injections were done with 40 mg in each side. Patient was placed on vigorous mouth opening exercises using Therabite devise. Patient responded well to the treatment regimen and maintained mouth opening at 30 mm after 12 months of treatment with good ability to have a satisfactory masticatory function (Figure 5). 

## 3. Discussion

Oral submucous fibrosis is a well-recognized potentially premalignant lesion of the oral cavity. Oral submucous fibrosis is also a common disease in countries where betel nuts are chewed habitually. It is believed to have multiple causes. Local irritants (betel nuts, tobacco, and spicy food), general nutrition, and vitamin deficiencies are considered to risk factors for oral submucous fibrosis [5]. 

Clinically, the earliest sign of oral submucous fibrosis is mouth soreness with constant burning upon eating spicy foods. Oral submucous fibrosis is clinically divided into 3 stages: stomatitis, fibrosis, and sequelae [6]. During the stomatitis phase, the oral mucosa contains areas of erythema in which vesicles appear. These vesicles later rupture producing ulcers that heal via fibrosis. The primary presenting complaint in this disease is progressive trismus from fibrosis of the buccal submucosa.

Treatment is based on severity of disease. Typically, if the disease is noted before development of trismus, cessation of the betel habit will often resolve the disease. Once trismus has developed and disease is now considered mild to moderate, oral submucous fibrosis is irreversible disease, with the goal of medical and surgical therapy to maintain oral function and limit progression of disease. Treatment at this stage is focused on restoring mandibular range of motion, oral cancer surveillance, and cessation of betel nut habit. Physical therapy combined with medical treatment is often utilized. Medical therapy in the United States is limited to weekly submucosal injections of steroids to limit progression of oral submucous fibrosis [7]. For cases in which initial surgical intervention is unsuccessful resulting in recurrent trismus usually secondary to lack of compliance with physical therapy or less commonly from shrinkage of the skin graft or alloplastic graft a more aggressive surgical therapy is indicated. Again, through excision of any fibrous bands intraorally, repeated masticatory muscle myotomy is required. Often in this situation, a larger soft tissue buccal defect is created needing large soft tissue reconstruction. This can include a temporalis pedicled flap, superficial temporalis fascia pedicled flap, or a radial forearm free flap combined with split thickness skin graft coverage [8, 9]. 

Steroids and especially glucocorticoids were first used in the treatment of oral submucous fibrosis and were extensively used in the past several decades because of their anti-inflammatory property. Cytokines and growth factors produced by inflammatory cells can promote fibrosis by inducing a proliferation of fibroblasts, upregulating collagen synthesis and downregulating collagenase production. Several glucocorticoids were used such as short-acting drugs (hydrocortisone), intermediate-acting drugs (triamcinolone), and long-acting drugs (betamethasone and dexamethasone). Glucocorticoids exert their anti-inflammatory activity by inhibiting the generation of inflammatory factors and increasing the apoptosis of inflammatory cells. They partially relieved patients of their symptoms at an early stage of oral submucous fibrosis as confirmed in many studies [10, 11]. Steroids were less useful in reversing he abnormal deposition of fibrotic tissues and thus this treatment was always associated with a high incidence of relapse. 

Several emerging advances were made past few years for the management of oral submucous fibrosis with promising results. Further studies recognized that proliferative fibroblasts, inactive collagenase, and the inhibited fibrinolytic system contributed to the initiation and development of oral submucous fibrosis [12, 13]. In a controlled clinical trial, H. J. Lin and J. C. Lin found that intralesional injections of collagenase resulted not only in significant improvement in mouth-opening but also in a striking reduction of hypersensitivity to spices, sour, cold, and heat. These results indicated that collagenase treatment was approximately fivefold more effective than triamcinolone diacetate [14]. Hyaluronidase also showed a much quicker effect in ameliorating the burning sensation and painful ulceration than did dexamethasone, though the effect was short-term [15]. Chymotrypsin, an endopeptidase, hydrolyzes ester and peptide bonds. It was also used as a proteolytic and anti-inflammatory agent in the treatment of oral submucous fibrosis [16].

Yen was the first to succeed in coveting the buccal defect with a split-thickness skin graft in treating a case of oral submucous fibrosis [17]. Kavarana and Bhathena filled the defect after sectioning of fibrous bands with 2 inferiorly based nasolabial flaps and division of the pedicle after 3 weeks they observed average mouth opening of 2.5 cm with acceptable external scars [18]. R. M. Borle and S. R. Borle reported disappointing results with skin grafting to cover the raw area and used tongue flap to cover the defect [19]. Khanna and Andrade reported the incidence of shrinkage, contraction, and rejection of split skin graft as very high leading to poor oral condition with recurrence in 12 cases [20]. Mehrotra et al. present a case series of 100 patients where they compared buccal fat pad graft, tongue flap, nasolabial fold flap, and split skin graft for correction of mucosal defect created after incising the fibrous bands. Esthetics and function achieved with split skin graft were good but showed some degree of relapse due to contracture of the graft. They found that buccal fat pad rotation was superior to other procedures [21].

Microscopically diffuse fibrosis in the submucosa with a chronic inflammatory infiltrate is the hallmark of oral submucous fibrosis. Atrophic changes in the mucosal layer include thinning of the epithelium, loss of rete ridges, sawtoothing, and liquefaction degeneration of the basal layer. Pigmentation is incontinence; Isaac et al. found that pigmentation was present in 62% of the 35 specimens they studied [22]. Areas of ulceration are usually seen replaced by granulation tissue. Fibrosis in the submucosa can be classified as mild if early fibrosis was present, moderate if diffuse fibrosis was seen, and severe if there was diffuse fibrosis with hyalinization and atrophic changes in minor salivary glands and skeletal muscle. Chronic inflammatory cells (i.e., lymphocytes, plasma cells, and macrophages) are also seen. As reported in the literature, the risk of oral cancer developing in oral submucous fibrosis is 7.6% over a period of 10 years [23]. Daftary reported that on followup, one third of his patients suffering from oral submucous fibrosis ultimately developing oral cancer [24]. 

In summary all available treatments for oral submucous fibrosis are basically palliative in nature. Surgical procedures are indicated only in cases with advanced disease. A safe and simple mode of treatment such as steroid or enzymes injection along with proper habit restriction should be selected in the earlier stages of the disease. Early detection is key toward better prognosis and outcome.
